# Constraints on the deformation of the vibrissa within the follicle

**DOI:** 10.1371/journal.pcbi.1007887

**Published:** 2021-04-01

**Authors:** Yifu Luo, Chris S. Bresee, John W. Rudnicki, Mitra J. Z. Hartmann

**Affiliations:** 1 Department of Mechanical Engineering, Northwestern University, Evanston, Illinois, United States of America; 2 Interdepartmental Neuroscience Program, Northwestern University, Evanston, Illinois, United States of America; 3 Department of Civil and Environmental Engineering, Northwestern University, Evanston, Illinois, United States of America; 4 Department of Biomedical Engineering, Northwestern University, Evanston, Illinois, United States of America; University of California San Diego, UNITED STATES

## Abstract

Nearly all mammals have a vibrissal system specialized for tactile sensation, composed of whiskers growing from sensor-rich follicles in the skin. When a whisker deflects against an object, it deforms within the follicle and exerts forces on the mechanoreceptors inside. In addition, during active whisking behavior, muscle contractions around the follicle and increases in blood pressure in the ring sinus will affect the whisker deformation profile. To date, however, it is not yet possible to experimentally measure how the whisker deforms in an intact follicle or its effects on different groups of mechanoreceptors. The present study develops a novel model to predict vibrissal deformation within the follicle sinus complex. The model is based on experimental results from a previous *ex vivo* study on whisker deformation within the follicle, and on a new histological analysis of follicle tissue. It is then used to simulate whisker deformation within the follicle during passive touch and active whisking. Results suggest that the most likely whisker deformation profile is “S-shaped,” crossing the midline of the follicle right below the ring sinus. Simulations of active whisking indicate that an increase in overall muscle stiffness, an increase in the ratio between deep and superficial intrinsic muscle stiffness, and an increase in sinus blood pressure will all enhance tactile sensitivity. Finally, we discuss how the deformation profiles might map to the responses of primary afferents of each mechanoreceptor type. The mechanical model presented in this study is an important first step in simulating mechanical interactions within whisker follicles.

## Author summary

Many mammals rely on whiskers as a mode of tactile sensation, especially when exploring in darkness. Active, rhythmic protraction and retraction of the whiskers, commonly referred to as “whisking,” is observed among many whisker specialist animals. During whisker-based sensing, forces and moments generated by external stimuli are transmitted to the base of the whisker shaft inside the follicle. Within the follicle, the interaction between the whisker’s deformation and the surrounding tissue determines how different groups of mechanoreceptors will deform, thereby transducing the mechanical signals into electrical signals. However, it is not yet possible to experimentally measure this interaction *in vivo*. We therefore created a mechanical model of the follicle sinus complex to simulate whisker deformation within the follicle resulting from external whisker deflection. Our results provide the first estimate of whisker shape as it deforms in the follicle, during both passive touch and active whisking. In turn, these shape estimates allow us to predict how the whisker will deform against different types of mechanoreceptors at different locations within the follicle. In addition, we find that both intrinsic muscle contraction and an increase in blood pressure will improve the tactile sensitivity of the whisker system.

## Introduction

Nearly all mammals have an extensive vibrissal (whisker) system [1], and many are specialized to actively gather tactile information from the environment [2–4]. Unlike an insect antenna, a whisker has no mechanoreceptors along its length. Instead, external mechanical stimuli are transmitted to a richly innervated follicle at the whisker base [5–10], where mechanoreceptors transduce the mechanical information into electrical signals [11,12]. Therefore, in order to understand how an animal detects, localizes, and perceives a whisker-based tactile stimulus, it is essential to understand how deformation of the whisker outside the follicle causes deformation of the whisker inside the follicle.

Whisker and follicle systems are thought to be traits of very basal mammals [13–15], or even the mammal-like reptile cynodonts [16–18], and whisker anatomy is strikingly similar for multiple species across a broad swath of the mammalian family tree. A list of these species helps impart an appreciation for the remarkable range of animals that share similar follicle characteristics and for the importance of this sensing modality in mammals: the rakali (water rat; *Hydromys chrysogaster*) [19], naked mole-rats (*Heterocephalus glaber*) [20], tree squirrels (*Sciurus vulgaris*) [21], shrews (*Sorex araneus*) [22], rock hyrax (*Procavia capensis*) [23], tammar wallaby (*Macropus eugenii*) [24], manatee (*Trichechus manatus*) [25,26], harbor seal (*Phoca vitulina*) [27], ringed seal (*Pusa hispida*) [28], California sea lion (*Zalophus californianus*) [29], sea otter (*Enhydra lutris*) [30], bearded seal (*Erignathus barbatus*) [31], Eurasian otter and pole cat (*Lutra lutra* and *Mustela putorius*) [32], rats and cats (*Rattus norvegicus* and *Felis catus*) [11].

Across these species, lengthwise cross-sectioning of each follicle is near-cylindrical (bearded-seal, pole cat, ringed seal, sea otter, and shrew), ovular (rat, cat, rock hyrax, wallaby, and manatee), or can resemble an inverted vase (squirrel). Regardless of shape, however, all follicles are densely packed with mechanoreceptors, often including Merkel, lanceolate, and club-like endings, and all contain one or more blood sinuses, which have been postulated to help regulate sensor sensitivity based on variations in blood pressure [33–36].

An animal’s perception of a tactile stimulus will be determined by how these mechanoreceptors transduce mechanical deformation into neural signals, which will in turn be determined by how the vibrissa deforms within the follicle. Additionally, the particular profile of whisker deformation has the potential to actuate different populations of mechanoreceptors along the length of the follicle.

To begin to quantify the deformation of the whisker within the follicle, Whiteley et al. [37] recently performed an experiment to determine how the internal follicle tissue at the ring sinus (RS) level deformed in response to a vibrissal deflection. With the caveats that this experiment was performed *ex vivo*, and that it examined only a small region of the whisker follicle sinus complex (FSC), the data from Whiteley et al. [37] provide ground truth measurements of the tissue displacements that result from passive whisker deformation relative to the follicle. These data thus provide a starting point for predicting more complex whisker-follicle interactions.

The present study was undertaken to investigate the deformation profile of the whisker within the follicle. In the present study, we develop a mechanical model of the FSC that replicates the deformation profile observed in passive conditions [37] and examine features that change during active whisking. These profiles allow us to predict how the whisker will deform against different types of mechanoreceptors at different locations within the follicle. Results also show that active muscle contraction, as well as blood pressure increases during the arousal concomitant with active exploration, may both help enhance tactile sensitivity.

## Materials and methods

### Ethics statement

All experiments involving animals were approved in advance by the Institutional Animal Care and Use Committee of Northwestern University.

### Anatomical experiments to estimate tissue stiffness along the follicle length

To obtain estimates of tissue stiffness within the follicle, we sectioned four mystacial pads of three adult (3–8 months), female, Long Evans rats (*Rattus norvegicus*). After use in unrelated electrophysiology experiments, rats were perfused with 1x phosphate-buffered saline solution (PBS) with 10 units/ml heparin and then with HistoChoice. The mystacial pad tissue was dissected away from the underlying bone and placed in 100% HistoChoice overnight. After 24 hours, tissue was sequentially cryoprotected in 10%, 20%, and 30% sucrose in PBS, each until osmotic pressure was equalized, as indicated by the tissue resting on the bottom of the vial. Tissue was then flash-frozen in Optimal Cutting Temperature compound (Tissue-Tek O.C.T., Sakura Finetek) on a level aluminum block partially submerged in liquid nitrogen, and sectioned at 20 microns on an upright freezing microtome.

Tissue sections were mounted on gelatin coated slides using a 4% paraformaldehyde solution for 15 min, and then permeabilized with acetone for 5 min. Sections were washed, bleached, and stained in Mallory’s Phosphotungstic Acid Hematoxilin (PTAH) [38], washed again and dehydrated; stained in 0.1% Fast Green in ethanol; and finally washed, cleared, and placed under cover slips. Fast Green stains collagen blue-green, while PTAH stains muscle striations purple-blue and many tissues (including collagen) various shades of red-pink. When we double-stained for collagen and muscle, the pink PTAH pigments were washed out with ethanol and the collagen was re-stained with Fast Green to achieve darker and more distinct color. Each slide-mounted section of a whole pad was placed under a Zeiss Opmi 6-CFC dissecting microscope. Photomicrographs were taken at 8x magnification with a Canon Digital Rebel camera.

### Overview of an *ex vivo* whisker deflection experiment

To constrain some model parameters, we used data from a study that experimentally quantified tissue deformation in an *ex vivo* preparation [37]. In these experiments, the displacement of tissue internal to the follicle near the RS level is imaged during external deflection of the whisker. An overview of the experimental procedure is provided here.

**Fig 1:**
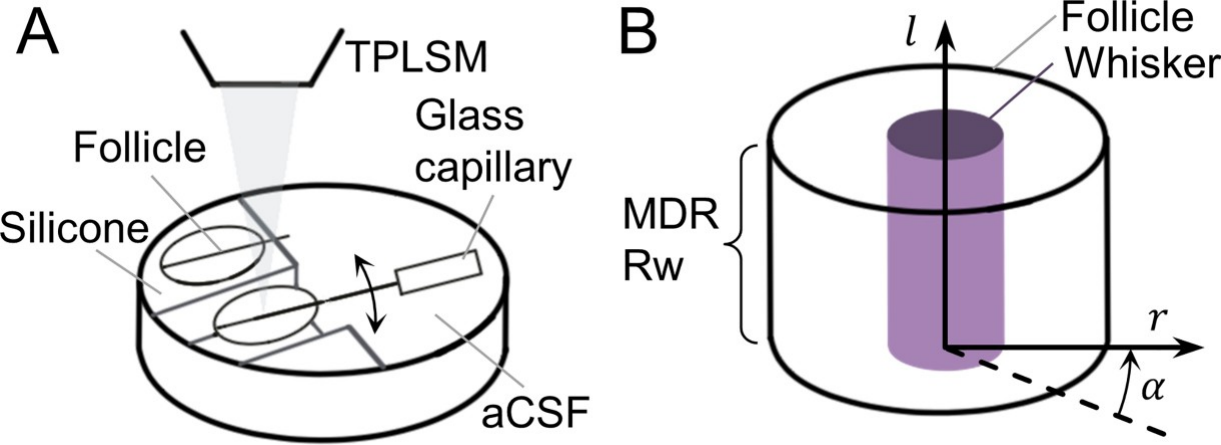
Schematic of the experimental procedure and coordinate systems used in Whiteley et al. [37]. (A) A row of follicles pinned to silicone base, immersed in artificial cerebrospinal fluid for two-photon imaging. A single whisker was deflected by displacing the glass capillary in rostral or caudal directions, while the dorsal surface of the follicle was exposed and imaged. TPLSM: two-photon laser scanning microscope; aCSF: artificial cerebrospinal fluid. (B) A cylindrical coordinate system was used for displacement analysis of the tissue between the whisker (purple cylinder) and the follicle. A section of the Merkel cell dense region at the level of the ringwulst was imaged. Radial distance (r) measures displacements perpendicular to the vibrissa; polar angle (α) measures displacements around the circumference of the vibrissa; and longitudinal distance (l) measures displacements along the vibrissa length. MDR: Merkel-cell dense region; Rw: ringwulst.

Briefly, Whiteley et al. [37] dissected the C-row of whiskers, suspended C1 horizontally in a petri dish, with flanking follicles supported by silicone, and deflected the C1 whisker proximally (~7mm) and horizontally with a high-resolution manipulator (Fig 1A). The wall of the actuated follicle at the level of the ringwulst was dissected, providing a window (~1×1mm^2^) for imaging the relative displacement of fluorescently labeled Merkel cells. Relative displacement was calculated as the difference between pre-deflection position of a Merkel cell, and its position at the peak of whisker deflection, from z-stacks of two-photon images. Displacement was considered using a cylindrical coordinate system within the follicle consisting of 3 dimensions: radial (displacement perpendicular to the whisker), longitudinal (displacement along the whisker), and polar (rotation around the whisker, clockwise from dorsal axis being 0°) (Fig 1B). Whiteley et al.’s results indicated that the whisker at the RS level moves to the opposite side of the follicle, in the direction of deflection. Total displacement in the three dimensions was 4.8μm. Finally, no sign change of radial displacement was identified in the observed window.

### A beam-and-spring model for the vibrissa displacement in the FSC

**Fig 2:**
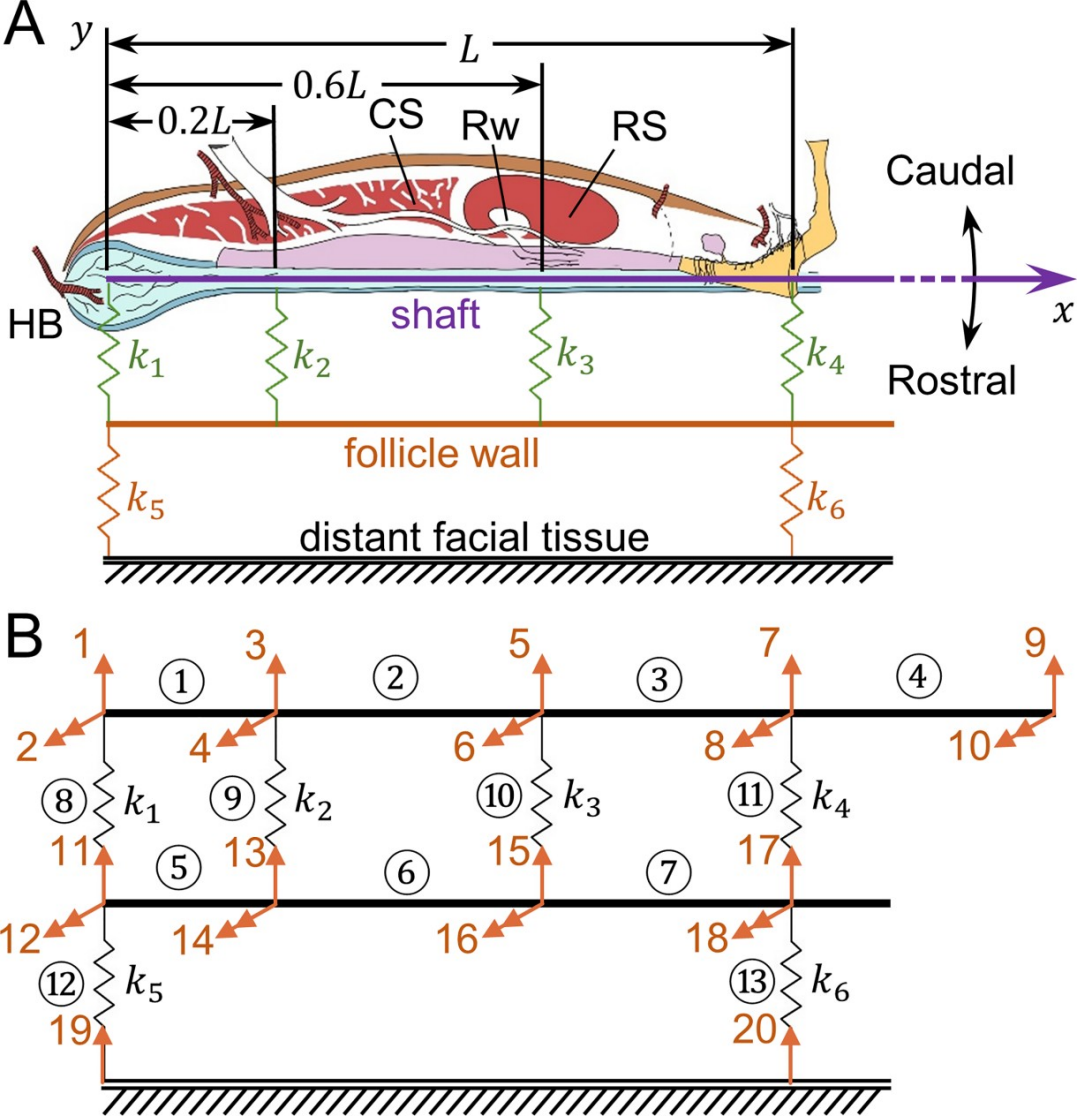
The mechanical formulation of the beam-and-spring model for the FSC. (A) The beam-and-spring model of the vibrissa and tissue. The tissue inside the follicle is modeled by four internal springs (k_1_, k_2_, k_3_, and k_4_), colored in green and placed at anatomically relevant locations. The tissue outside the follicle is modeled by two external springs (k_5_ and k_6_), colored in orange. Although the springs are *illustrated* only on one side, they *act* on both sides, or more precisely, all around the shaft. The vibrissa is represented as a purple line, and the follicle wall is an orange line. The follicle system is supported by the rest of the tissue on the face, considered to be far away and thus indicated as ground. The overall length of the follicle is L, from the base to apex. (B) The finite element definition of the vibrissa-follicle structure. The entire structure is modeled by 13 elements (beam segments and springs) denoted by black circled indices. There are a total of 20 degrees of freedom (DOFs) denoted by orange arrows and indices. Single headed orange arrows denote translations; double-headed orange arrows denote rotations. Elements 1~4 represent the segments of the vibrissa, elements 5~7 represent the segments of the follicle wall, and elements 8~13 represent six springs.

We created a beam-and-spring model (Fig 2A) to simulate deformation of the vibrissa in the follicle and the follicle in the tissue. Two beams and six springs were used.

The whisker is represented by a Bernoulli-Euler beam [39] and the follicle wall by a rigid beam. The tissue distribution internal to the follicle wall is modeled by four *internal springs* (k_1_, k_2_, k_3_, and k_4_), chosen at the locations of the hair bulb (HB), the cavernous sinus (CS), the RS, and the follicle entrance. These locations were chosen based on the approximation that material properties are similar within each of the three partitioned regions. The connective tissue and muscle outside of the follicle are represented by two *external springs* (k_5_ and k_6_), representing the locations of the two contact points of the intrinsic muscles at the top and bottom of the follicle, respectively. The adjacent follicles and distant facial tissue are indicated as rigid ground in the schematic, and their elastic properties are accounted for in the choices of k_5_ and k_6_. This approximation is appropriate because in all experiments and simulations presented in this work, only a single follicle is deflected at a time. Simulating simultaneous deflection of multiple whiskers is a topic for future work.

We emphasize that the schematic is not intended to imply that either tissue or muscle insertion points exist only on one edge of the follicle. Tissue surrounds the follicle and the whisker shaft on all sides. However, the model only simulates whisker deflection in the rostrocaudal direction (in line with the intrinsic muscles). In this model, complex mechanical effects, including normal and shear effects from other dimensions, are simplified to act only in the plane of whisker movement. In other words, the model is “pseudo-3D” since it allows movement only in one plane (2D), but incorporates tissue effects in all dimensions. Furthermore, placing springs on one side is mathematically equivalent to modeling the mechanics on both sides of the movement direction, because compression and tension can be modeled with the identical spring with a sign change.

Although this model is simplified, we emphasize that our goal is only to simulate the overall whisker deformation, not the exact internal tissue displacement and strain. The model captures the essential features of the follicle stiffness distribution and allows examination of a finite, but wide, range of spring stiffnesses. The model is well suited for finite element method analysis. The solution to this problem employs a standard approach of stiffness matrix assembly [40]. A proper decomposition of the structure for finite element analysis is shown in Fig 2B.

With these definitions, we define the global displacement matrix

$$d^{T}=[u_{1},\theta_{2},u_{3},\theta_{4},u_{5},\theta_{6},u_{7},\theta_{8},u_{9},\theta_{10},u_{11},\theta_{12},u_{13},\theta_{14},u_{15},\theta_{16},u_{17},\theta_{18},u_{19},u_{20}]$$ (1)

where the translational displacements and rotational displacements are denoted by u and θ, respectively.

The stiffness matrices for elements 1 to 7 are

$$K^{\left(i\right)}=\begin{array}{cc} \left[\begin{array}{cccc} \frac{12E_{i}I_{i}}{L_{i}^{3}} & \frac{6E_{i}I_{i}}{L_{i}^{2}} & -\frac{12E_{i}I_{i}}{L_{i}^{3}} & \frac{6E_{i}I_{i}}{L_{i}^{2}}\\ \frac{6E_{i}I_{i}}{L_{i}^{2}} & \frac{4E_{i}I_{i}}{L_{i}} & -\frac{6E_{i}I_{i}}{L_{i}^{2}} & \frac{2E_{i}I_{i}}{L_{i}}\\ -\frac{12E_{i}I_{i}}{L_{i}^{3}} & -\frac{6E_{i}I_{i}}{L_{i}^{2}} & \frac{12E_{i}I_{i}}{L_{i}^{3}} & -\frac{6E_{i}I_{i}}{L_{i}^{2}}\\ \frac{6E_{i}I_{i}}{L_{i}^{2}} & \frac{2E_{i}I_{i}}{L_{i}} & -\frac{6E_{i}I_{i}}{L_{i}^{2}} & \frac{4E_{i}I_{i}}{L_{i}} \end{array}\right] & \begin{array}{c} a_{i}\\ b_{i}\\ c_{i}\\ d_{i} \end{array}\\ \begin{array}{cccc} a_{i} & b_{i} & c_{i} & d_{i} \end{array} & \end{array},\;i\in \{1,2,3,4,5,6,7\},$$ (2)

where E is Young’s modulus, I is the moment of inertia (= πR^4^/4, R: vibrissa base radius), and L is the length of the corresponding element. The indices [a_i_, b_i_, c_i_, d_i_] below and to the right of the matrix correspond to the indices of the DOFs for the i-th element when it is added to a global stiffness matrix. For example, [a_1_, b_1_, c_1_, d_1_] = [1,2,3,4] for element 1.

Similarly, the stiffness matrices for elements 8 to 13 are

$$K^{\left(i\right)}=\begin{array}{cc} \left[\begin{array}{cc} k_{i-7} & -k_{i-7}\\ -k_{i-7} & k_{i-7} \end{array}\right] & \begin{array}{c} a_{i}\\ b_{i} \end{array}\\ \begin{array}{cc} a_{i} & b_{i} \end{array} & \end{array},\;i\in \{8,9,10,11,12,13\},$$ (3)

where k_i-7_ are the spring constants for the i-th element. The indices [a_i_, b_i_] below and to the right of the matrix correspond to the indices of the DOFs at each end of the i-th element when it is added to a global stiffness matrix. For example, [a_10_, b_10_] = [5,15] for element 10.

When the global stiffness matrix is constructed, linear shape functions reflect the assumption of constant Young’s modulus along the vibrissa. The global stiffness matrix is a 20-dimensional sparse square matrix, assembled by adding up values from local stiffness matrices sharing the same indices pair defined previously, or by using the scatter operator **L**^(i)^, dependent on indices for corresponding DOFs, to stack the local stiffness matrices to a global sparse matrix. The result is

$$K=\sum_{i=1}^{13}L^{(i)T}K^{\left(i\right)}L^{\left(i\right)}$$ (4)

In addition to the natural boundary condition that u_19_ = u_20_ = 0, we also apply the essential boundary condition that θ_8_ = -10°, to simulate a 10° deflection of the vibrissa. By using penalty method [41,42], the overall stiffness matrix and force matrix is given by

$$K^{tot}=K+diag(0,0,0,0,0,0,0,\beta ,0,0,0,0,0,0,0,0,0,0,\beta ,\beta )$$ (5)

$$f={\left[0,0,0,0,0,0,0,\beta {\bar{\theta }}_{8},0,0,0,0,0,0,0,0,0,0,\beta {\bar{u}}_{19},\beta {\bar{u}}_{20}\right]}^{T}$$ (6)

where β is a very large number usually 10^7^ to the average K(ii) to enforce the natural boundary conditions. In our calculation, we choose β = 10^7^E (Young’s modulus).

The nodal displacements are then related to the nodal forces by

$$d=K^{-1}f$$ (7)

The shear force V(x) along the vibrissa is simply the cumulative sum of the forces caused by the deformation of those springs. For the Euler-Bernoulli beam, the deflection of the beam u(x) is fully described by the equation

$$EI\frac{d^{3}u(x)}{dx^{3}}=V(x),x\in [0,L]$$ (8)

The Young’s modulus (E) for the vibrissa is estimated to be 3.5GPa based on several studies [43,44], the follicle length (L) is measured to be ~1mm, and the diameter of the vibrissa near its base is assumed to be ~150μm [11]. Note that because the displacements can be expressed in a nondimensional form, change in these estimates simply scales the magnitude of displacements. The boundary conditions are the displacement on both ends and the bending moment on a solved end. In particular, the bending moment for the base of the follicle (free end) is M(0) = 0.

**Fig 3:**
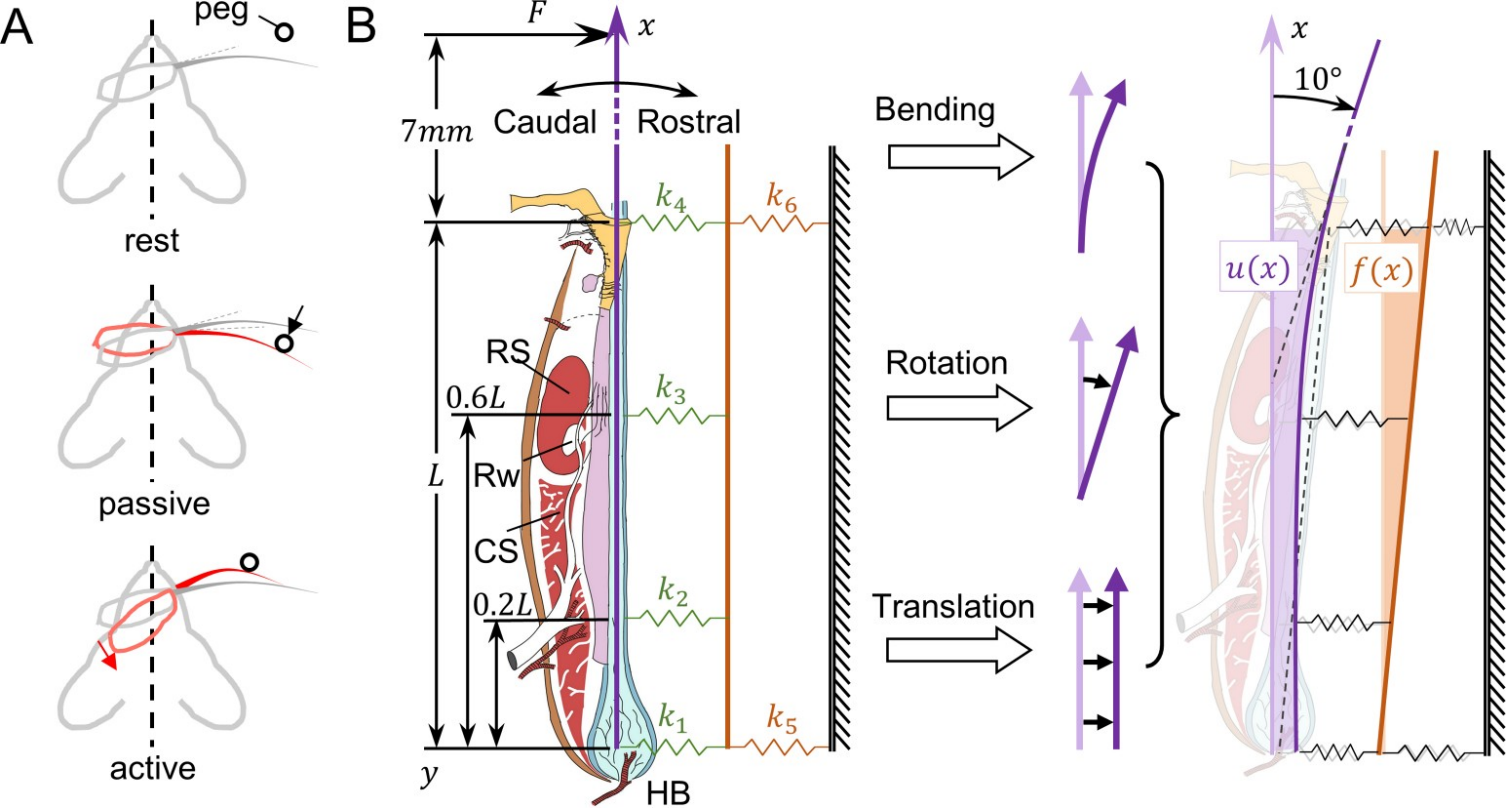
Relative and absolute displacements during whisker deflection. (A) Illustrations demonstrating mechanical differences between passive touch and active whisking. **Top:** the whisker and follicle at their resting locations, undeflected. **Middle**: during passive touch, the whisker is deflected by a peg, leading to movement of the loosely-held follicle. **Bottom**: during active whisking, the follicle is driven by contracted muscle, which is stiff. In both cases, the follicle moves upon deflection. (B) Modeling displacement caused by external deflection of the whisker. **Left**: The beam-and-spring model of the vibrissa and follicle. The undeflected vibrissa lies along the x-axis. Four springs (k_1_, k_2_, k_3_, k_4_) connect the vibrissa (purple) to the follicle wall (orange). Two springs (k_5_, k_6_) connect the follicle wall to distant facial tissue (ground). **Middle**: The deformation of the vibrissa in response to an external applied force F is composed of three elemental components: bending, rotation, and translation. Because the follicle wall is modeled as a rigid beam (i.e., much stiffer than the vibrissa), it displaces only by translation and rotation. **Right**: Schematic after a 10° rostral rotation of the vibrissa. The absolute displacement u(x) is shown in light purple. The follicle wall displacement f(x) is shown in light orange.

Upon deflection of a whisker, the follicle will not stay stationary relative to the animal’s head. We contrast differences and similarities of follicle movement between passive touch and active whisking qualitatively in Fig 3A. The follicle will be driven either by the deflected whisker (passive touch), or by actuated intrinsic muscle (active whisking).

We define the following different quantifications of displacement relevant to this study by showing an example of 10° whisker deflection by illustration (Fig 3B). The *absolute displacement* u(x) of the vibrissa is the difference between its deflected position and its original undeflected position for all points on the vibrissa. The deflection of a vibrissa includes bending, rotation, and translation, all of which contribute to the absolute displacement u(x) of the whisker. S1 Fig contains a simplified and intuitive version of Fig 3B. The *follicle wall displacement* f(x) is similarly defined for the follicle wall. Finally, the *relative displacement* r(x) of the vibrissa is defined as the difference between u(x) and f(x). The relative displacement is more relevant than absolute displacement because it determines how the mechanoreceptors along the whisker length interact with the internal tissue.

## Results

### Overview: Novelty of results and limitations on their interpretation

The present work provides the first estimate of the shape of the whisker as it deforms in the follicle, during both passive touch and active whisking. These shape estimates in turn allow us to predict how the whisker will push into (and pull against) different types and groups of mechanoreceptors at different locations within the follicle, and these ideas are elaborated extensively in the Discussion. The model also makes predictions for how muscle and tissue stiffness, as well as blood pressure in the RS, will affect the whisker’s deformation profile in the follicle, and thus ultimately how they will affect mechanoreceptors and the rat’s tactile sensitivity.

All results of the present work should be interpreted in a semi-quantitative manner. The model can predict the order of magnitude of relative displacements of the whisker shaft at different locations within the follicle. In addition, the present results apply only to quasi-static conditions, and thus cannot be applied to whisker collisions, vibrations, texture exploration, or airflow.

### Parameter constraints and optimization

Although the structure of the mechanical model has been established based on anatomy (Fig 3), the values of the spring constants representing the tissue stiffness are as yet unconstrained. To constrain some of the stiffness values in the model we used prior experimental work as well as a new anatomical analysis.

#### Skin stiffness imposes constraints on k_4_, k_5_, and k_6_

We begin by constraining k_4_, k_5_, and k_6_. The two external springs (k_5_ and k_6_) represent the muscle attachment and model the rotation and the translation of the follicle within the tissue. Consider the case that the whisker is deflected when the intrinsic muscle outside the follicle is relaxed (e.g., when the animal is anesthetized, resting, or unprepared for an external stimulus). In this case, relaxed muscle together with other connective tissue is representative of the overall skin stiffness (8MPa for mouse [45]). Therefore, we approximated the overall skin stiffness in our model as the sum of k_5_ and k_6_ by multiplying the elastic modulus E_skin_ by the follicle length:

$$k_{5}+k_{6}\approx E_{skin}\cdot L=8MPa\times 1mm\approx 10^{4}N/m$$ (9)

Notice that the ratio of k_5_ and k_6_ will depend on the exact state of the intrinsic muscle; this ratio will become important later in our analysis of active vs. passive deflections. In the simulations that follow, we used a ratio of k_6_/k_5_ = 7/3, unless otherwise indicated. This value was chosen to reflect the relatively stiffer skin tissue (near k_6_) compared to the compliant fat tissue deeper in the mystacial pad (near k_5_). Although 7/3 is only an approximation, later results will demonstrate that this ratio can vary widely without significantly changing the shape of the deformation profile.

To constrain the value of k_4_, the spring at the follicle entrance, we noted that previous work has indicated that the vibrissa tends to be stiffly clamped as it enters the follicle [46]. It is clear from the videos associated with this earlier study that the vibrissa displaces very little relative to the follicle at its entrance. To ensure such a rigid vibrissal-follicle junction, k_4_ should be much larger than the unactuated intrinsic muscle stiffness represented by the sum of k_5_ and k_6_. A factor of 100 is sufficient to prevent translation (and permits only rotation) at the follicle entrance:

$$k_{4}\geq 100(k_{5}+k_{6})=10^{6}N/m$$ (10)

#### Follicle anatomy imposes constraints on k_1_, k_2_, k_3_, and k_4_

**Fig 4:**
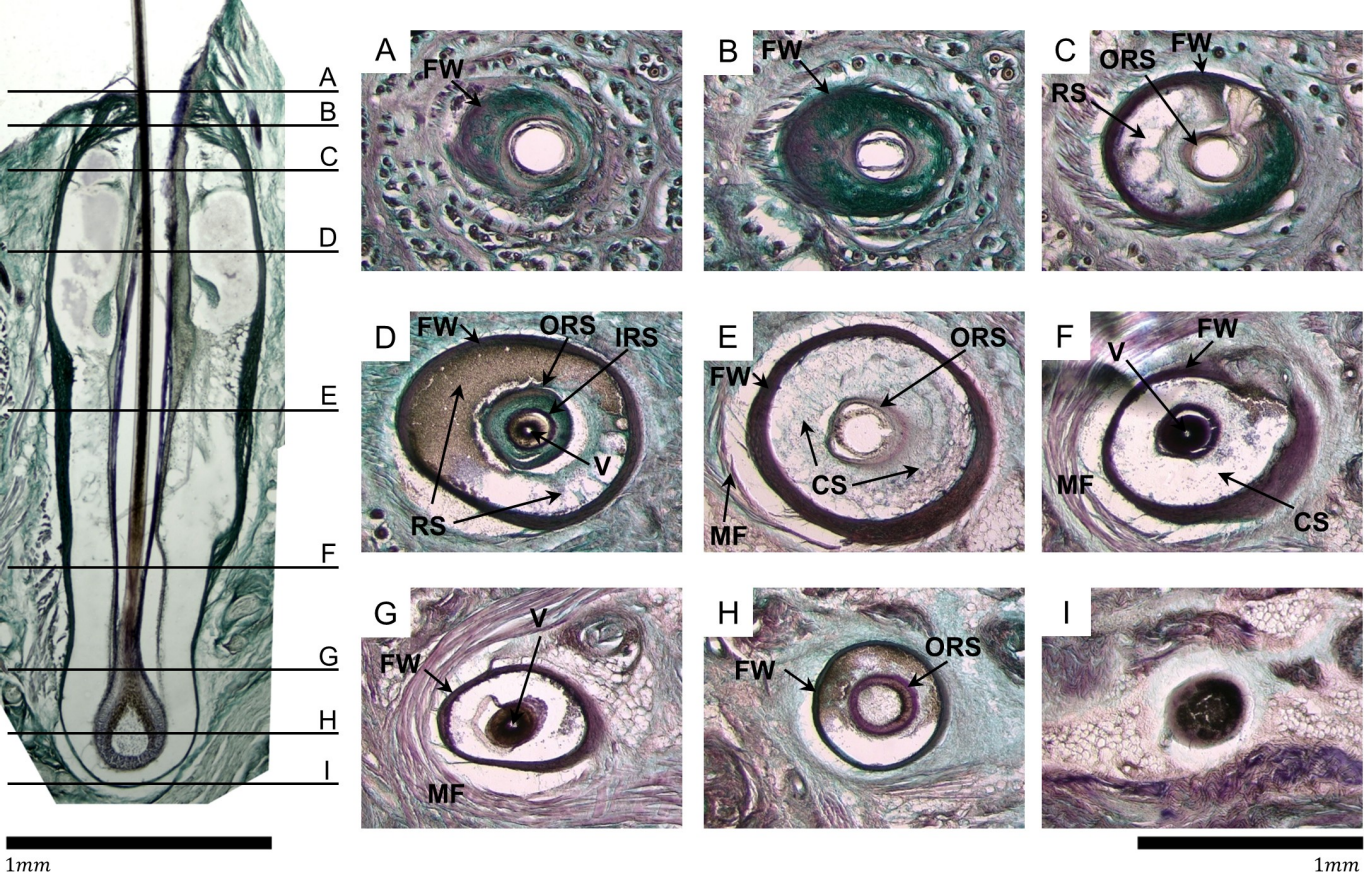
Images of horizontal (lengthwise) and parasagittal (cross-sectional) FSC sections permit estimation of relative tissue stiffness. All images were taken at 4x. **Left:** The image shows a lengthwise cross-section of the A1 vibrissal FSC, assembled from multiple tiled images. Rostral is to the left. The slice does not pass exactly through the central axis, so the vibrissa (the continuous medial dark vertical line) is actually thinner than the full whisker diameter, and no medulla can be observed. The large space free of tissue observed around the vibrissa is an artifact of tissue preparation and was not represented in the model (see text for details). This panel serves only as a depth reference for the right panels and was not used for stiffness estimation. Horizontal black lines, labeled A–I, from superficial to deep represent the approximate levels at which the cross-sections (right 3x3 panel) were taken, on an equivalent follicle. Scale bar: 1 mm. **Right:** Cross sections of a single C3 vibrissa from superficial (section A) to deep (section I). Brightness, contrast, and magenta (R: 255, G: 0, B: 255) saturation were globally increased during post-processing. The vibrissa is present in sections D, F, G, and H, but has fallen out of the tissue in the remaining sections and is observed as a white oval. The whisker often falls out during tissue preparation because the keratin from which it is composed is highly cross-linked, and so does not have many available binding sites for fixative. The whisker in section H is diffuse and appears to be an empty space at 4x, but cells are observable at higher magnification, consistent with other descriptions of the HB and papilla [11]. **A, B:** A white oval where the vibrissa would be tightly held by the follicle wall (FW) is in the center of the image, and the follicle itself is also held tightly in the skin by the tissue. **C:** The outer root sheath (ORS), a membrane surrounding the vibrissa, is observed as a dark oval. Because the follicle has been sectioned at a slight angle, the RS (slightly deeper in the follicle) is observable on the rostral side. **D:** At the level of RS, most of the space inside the follicle is occupied by blood (brown) or empty space (white). The ORS and inner root sheath (IRS) can both be observed as dark ovals. The vibrissa (V) is held closely against the IRS, though the outer layer of the hair shaft is not pigmented so appears very light white/gray. **E:** Leaving the RS, all internal tissue becomes less stiff, appearing less darkly stained. At the level of CS, the FW is very thick compared to the vibrissa. MF: muscle fiber. **F:** Medially to the trabecula-dense region of the CS the internal membranes become denser (darker) but the vibrissa shaft becomes more diffuse, with melanocytes (pigmented portion of the whisker) no longer segregated to the center of the shaft (more apparent at higher magnification). **G, H, I:** All surrounding tissue is much less dense towards the end of the follicle, near the HB level. The whisker is also less dense. By comparing across all sections (A–I) it can be observed that the follicle wall is thin near the apex, thicker in the middle, and thin again deep in the tissue. Scale bar: 1 mm.

Having established basic constraints for k_4_, k_5_, and k_6_, we next estimated the values for the three remaining internal springs (k_1_, k_2_, and k_3_). To estimate the internal tissue stiffness at different levels of the follicle, we carefully examined serial images of mystacial pad tissue sliced so as to reveal follicle cross-sections (Fig 4).

The follicle is composed two major structural proteins: keratin (stained pink in Fig 4) and collagen (stained green in Fig 4). Keratin has a higher stiffness and is more resistant to deformation and displacement. Collagen has a lower stiffness and is more elastic. Because all tissue sections were processed with identical sectioning and staining techniques, the images serve as indicators of relative stiffness at different levels of the follicle. We compared relative stiffness across sections by considering the relative amounts of keratin and collagen in each section, as well as the density of the tissue as indicated by the darkness of stains. All assessments were made by looking with the naked eye through the microscope. Three major inferences about relative stiffness at various levels within and without the FSC can be made from the images in Fig 4.

First, throughout the length of the follicle, the follicle wall is very darkly stained, indicating that it is stiffer than the other collagen (green-stained) tissues in the image. In addition, the relative ratio of keratin and collagen in the follicle wall does not appear to change, so the thickness of the follicle wall can be taken as a proxy for its stiffness. The follicle wall is relatively thin in sections A and B, increases in thickness through section F, and becomes thin again at the far end (sections G, H, I). Note that the follicle has been sectioned at a slight angle so the follicle wall is a bit thicker on the caudal side; this effect is particularly noticeable in sections C and F. This anatomy validates the modeling assumption made in *Materials and Methods* that the follicle wall is rigid compared to the tissue inside and outside (Fig 3).

Second, the stiffness of the tissue internal to the follicle generally decreases from superficial to deep. At the skin surface (sections A, B, represented by k_4_), the whisker (missing) is seen as a white oval, and is densely surrounded by darkly stained tissue. Consistent with previous studies [46], sections A and B show that the whisker is held tightly at the apex of the follicle. This effect is partially attributable to the intrinsic stiffness of the tissue and partially attributed to the small size of the opening at the apex of the follicle which restricts vibrissal displacement at the entrance. These features support the previous assumption that k_4_ should be large. In section C, the outer root sheath becomes visible as a dark band around the whisker (white oval), and the surrounding dense staining indicates that the whisker continues to be held tightly within the follicle. Starting with level D, near the RS (k_3_), the whisker begins to be less tightly held within the follicle, as indicated by the lighter staining between the outer root sheath and the follicle wall. Sections E and F show even weaker staining inside the follicle near the CS (k_2_), indicating a continuing decrease in stiffness. This trend continues through section G, which approaches the plate, a mat of connective tissue that loosely overlies the bone [47]. Finally, in sections H and I (k_1_) we see very diffuse keratinocytes that will become the whisker cortex, and the very end of follicle capsule. In these sections the tissue is more hydrated and less dense, and the whisker is quite loose within the follicle.

Third, the follicle is held fairly tightly within the skin in sections A, B, and C. In section C, the whisker is primarily surrounded by keratin (stained pink), which is less elastic and tougher than collagen. By section D, the surrounding matrix is primarily loosely coiled collagen (stained green) and a large crescent of pale green to white is visible around the follicle’s rostral edge. This lighter staining indicates that the follicle is held loosely in the skin. In section E, the first fibers of the sling muscle are visible as pink strands running across the rostral arc of the follicle, and again a space lies between the follicle wall and the muscle fiber, showing that there is not a lot of connective tissue anchoring the follicle to surrounding tissue. The muscle can slide across the follicle and the follicle can slide around in the skin very easily. In these images, we distinguish pink muscles and pink keratin based on the presence of muscle fibers under microscope. Sections G, H, and I, continue this trend, showing large white/light green rings/crescents around the follicle that indicate that it is not well anchored in the skin.

Together, the images of Fig 4 suggest that it is reasonable to assume that k_1_≤k_2_<k_4_, that k_1_≤k_3_<k_4_, and that k_5_<k_6_. We cannot infer anything about the relative values of k_2_ and k_3_.

Less pertinent to the purpose of tissue stiffness estimation, we note that there is large space free of tissue observed around vibrissa in the left panel of Fig 4. This large space around the whisker shaft occurs partially from tissue shrinkage during cryoprotection, and partially because the plane of section is off center, and so the image does not include the full width of the whisker shaft. Although this artifact is visible in the anatomical images, it has no implications for the model, because the model does not assume any space between the whisker and surrounding tissue. In other words, the space is an artifact, and we did not include this artifact in the model.

### The deepest internal spring k_1_ has a negligible effect on deformation near the RS

The previous section constrained values for the two external springs, k_5_ and k_6_, as well as the most superficial spring, k_4_. We have also constrained the relative relations for the internal springs k_1_, k_2_, and k_3_, but have left their exact values uncertain. In this section, we show that the effect of k_1_ is negligible regardless of the values k_2_ and k_3_, and therefore it can be removed from the next stage of simulation.

To bracket the possible range of whisker deformation profiles we first investigated how k_1_ affects the relative displacement, r(x), in the RS region. Specifically, we simulated the rostral deflection of a vibrissa, and observed the deformation profile of the whisker for different values of k_1_, under different combinations of k_2_ and k_3_.

**Fig 5:**
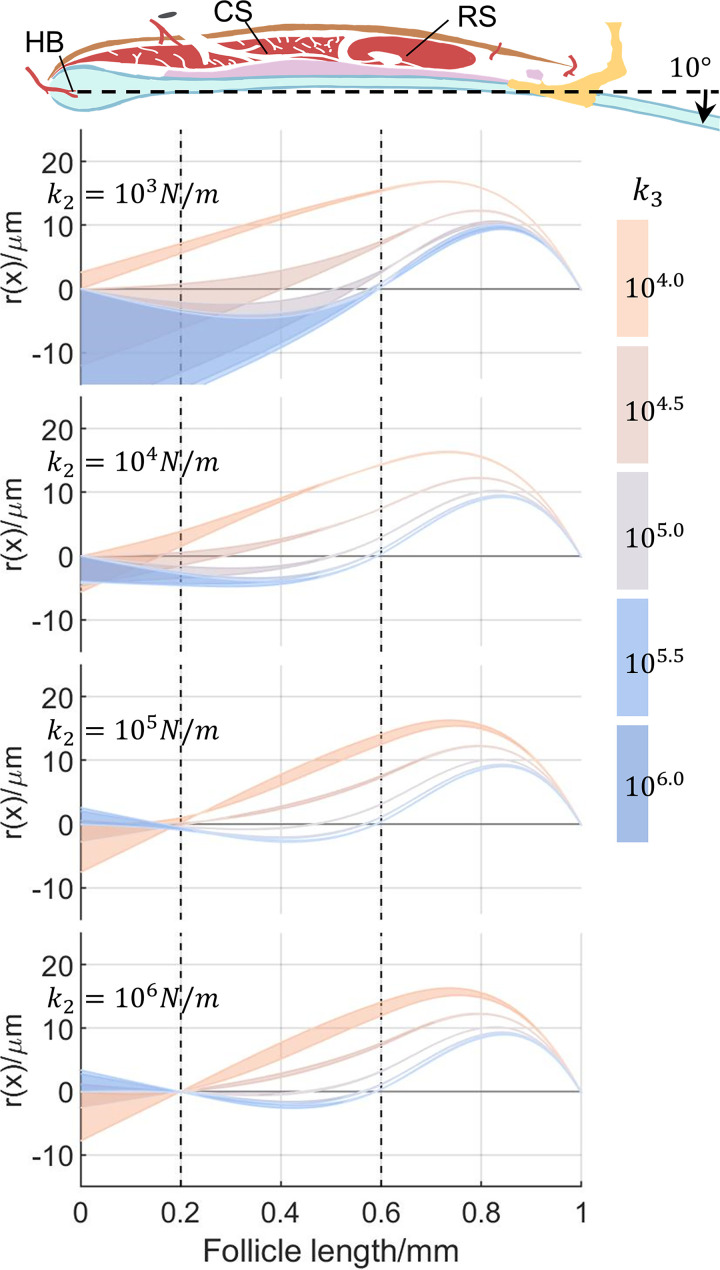
Varying k_1_ has a negligible effect on whisker deformation near the level of the RS. The schematic of the follicle at the top provides a visual reference for anatomical locations. Abbreviations: HB: hair bulb; CS: cavernous sinus; RS: ring sinus. The whisker is simulated to experience a 10° deflection for different combinations of spring constants (k_1_, k_2_, k_3_), and relative displacement r(x) is plotted against the follicle length. The aspect ratio of all plots has been exaggerated. From top to bottom, each of the four plots shows a different value of k_2_, increasing from 10^3^N/m to 10^6^N/m, logarithmically spaced. Within each plot, each color indicates a different value of k_3_, increasing from 10^4^N/m (orange) to 10^6^N/m (blue), logarithmically spaced. Each single shaded area represents the effect of varying k_1_ between 10^2^ and 10^6^N/m, for particular values for the other springs (k_2_ and k_3_). The width of each shaded area can be quite large at the level of the CS especially in the top two panels, but small (0.286**μ**m on average) at the level of the RS.

Fig 5 illustrates the different possible shapes of the relative displacement given a 10° deflection. The value of k_2_ changes across plots from top to bottom, and four distinct values of k_3_ are indicated as four different colors. For each k_2_ and k_3_ combination, changing k_1_ results in a shaded area bounded by two extreme deformation profiles. The width of the shaded area can be as large as 13.0μm microns near the CS (for the smallest value of k_2_). However, the width of the shaded area at the RS level is very small for all combinations of k_2_ and k_3_ (0.286μm in average), indicating that the effect of k_1_ is negligible in this region. Because our primary area of interest is around the RS in later stages of simulation, we set k_1_ to an intermediate value (10^3^N/m) to reduce the degrees of freedom in subsequent parameter tuning.

### The internal spring k_3_ has the largest effect on deformation around the RS

**Fig 6:**
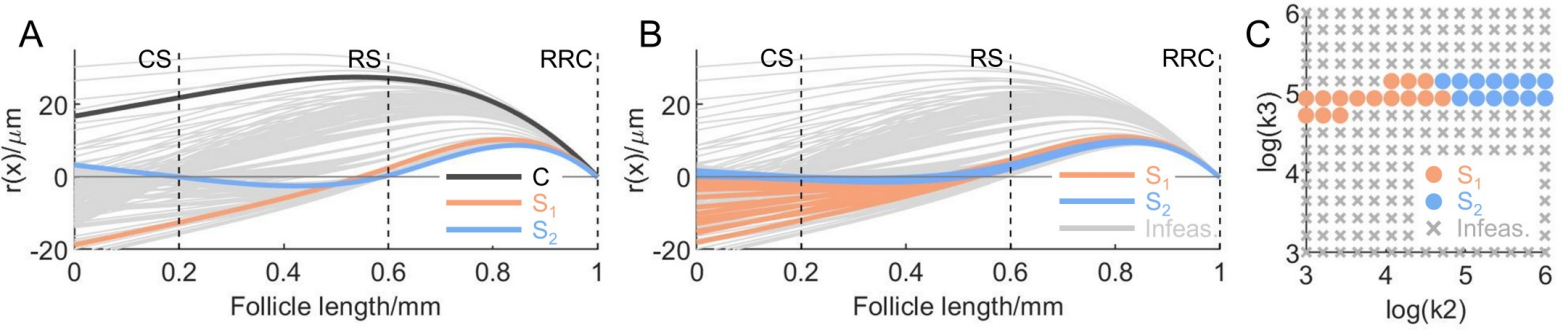
Although simulations across all possible (k_2_, k_3_) pairs permit both C-shaped and S-shaped deformation profiles, results from *ex vivo* experiments are most consistent with S-shaped profiles. (A) Different deformation profiles (r(x)) for different spring pairs (k_2_, k_3_) are shown as a cluster of gray curves. Some typical examples of the deformation profiles of the vibrissa are drawn in black (C-shape), orange (S_1_-shape), and blue (S_2_-shape). (B) Deformation profiles inconsistent with the *ex vivo* experimental data [37] are deemed infeasible and are drawn in gray. Feasible deformation profiles constrained by limiting the displacement at the RS level, r(x)|_x = 0.6mm_, to be smaller than 4.8μm are colored in orange and blue, depending on whether it is S_1_- or S_2_-shape. All profiles in both (A) and (B) have exaggerated aspect ratios for visual clarity. (C) The sampling space for (k_2_, k_3_) pairs is shown on a logarithmic scale bounded by [10^3^, 10^3^]N/m and [10^6^, 10^6^]N/m. The color scheme is the same as in A and B, and the space incompatible with the *ex vivo* data is marked with gray crosses. The space compatible with the *ex vivo* data is marked with orange or blue colored dots.

With the value of k_1_ now fixed, we examined the effect of different k_2_ and k_3_ values on the whisker deformation profiles. Depending on different (k_2_, k_3_) pairs, the deformation profile can take different shapes. Fig 6A shows all possible shapes that a vibrissa might take, with three typical shapes indicating three major categories of all deformation profiles, *C-shapes*, *S*_*1*_*-shapes*, and *S*_*2*_*-shapes*. These three different deformation profiles differ by the number of times they cross the x-axis (the resting position). Fig 6 confirms that the intermediate choice for k_1_ was appropriate, as these same three shapes were also apparent in Fig 5. In other words, the choice for k_1_ does not substantively restrict the range of possible deformations profiles of the whisker in the follicle.

The side(s) on which mechanoreceptors will be directly exposed to stretched or compressed tissue at different levels are determined by the locations of the x-axis crossings. The C-shaped profiles remain on a single side of the zero line, opposite the direction of vibrissa deflection. In contrast, S_1_-shapes cross the zero line once, near deeper levels of the follicle, below the RS. Many of the S_1_-shapes more closely resemble a “hook” or a cane than an “S.” S_2_-shapes cross the zero line twice, so they are deformed toward different sides of the follicle at different levels. Compared with the two S-shaped profiles, C-shapes have relatively low values of both k_2_ and k_3_. S_1_-shaped profiles have smaller values of k_2_ than do S_2_-shaped profiles.

To further constrain the internal spring constants k_2_ and k_3_, as well as the possible deformation profiles, we noted two features of the experiment by Whiteley, et al. [37]. First, the maximum tissue displacement near the RS relative to the follicle wall is calculated to be 4.8μm (see Methods). For small deflections, the maximum tissue displacement usually takes place at the leading edge (towards the direction of displacement) of the whisker during deformation. We therefore conservatively restricted the whisker displacement at the RS level to be smaller than 4.8μm. As will become clear, this numerical constraint has a large influence on the range of feasible profiles, and the consequences of relaxing it will be described in the *Discussion*. Second, there was no sign change of whisker displacement identified in the experiment. We therefore added the constraint that, within the window of the RS (x∈[0.55L, 0.65L]), the whisker must displace entirely towards a single side, opposite to the direction of whisker deflection.

With these two additional constraints imposed, we sampled the spring pair (k_2_, k_3_) drawn from a logarithmically spaced grid bounded by 10^3^N/m and 10^6^N/m in both dimensions, and plotted the relative deformation profiles (Fig 6B). As can be seen, when r(x)|_x = 0.6mm_ is constrained to be smaller than 4.8μm, the feasible deformation profile is limited to S_1_- or S_2_-shapes, and the C-shape is excluded. Consistent with the second constraint, all profiles deemed feasible in Fig 6B (both S_1_- and S_2_-shapes) indicate that the tissue is compressed on the side opposite to the deflection direction throughout the region from the RS to the rete ridge collar (RRC), and stretched elsewhere.

Fig 6C illustrates the (k_2_, k_3_) sample space, with S_1_- and S_2_-shaped profiles indicated using the same color scheme as in Fig 6B. The feasible space of (k_2_, k_3_) depends only weakly on k_2_, but strongly on k_3_. The value of k_3_ determines the whisker displacement around the RS level, and the value of k_2_ mostly determines whether the whisker takes an S_1_- or S_2_-shape.

Overall, the results shown in Fig 6 reveal two interesting features of vibrissal deformation in the follicle. First, because the spring at the entrance to the follicle is stiff, the model confirms a previous hypothesis [48] that a deflected vibrissa pivots about a fulcrum near the follicle apex. Therefore, in the superficial regions of the follicle, including the RRC and the RS, the vibrissa will deform in the direction opposite deflection. Although this result is not surprising from a mechanical perspective, it has important implications for the sensitivities of RS-Merkel and RRC-Merkel mechanoreceptors, both of which were recently shown to respond strongly in the (same) direction of vibrissal deflection (not opposite) [49]. Second, the model predicts that maximal deformation of the vibrissa will occur superficial to the mechanoreceptor-rich RS area. We note that this second result is largely due to the 4.8μm constraint imposed by the *ex vivo* experimental data. If this numerical constraint were relaxed, many of the C-shaped profiles would become feasible and much larger deformations in the RS area could occur. These ideas are elaborated further in the *Discussion*.

**Fig 7:**
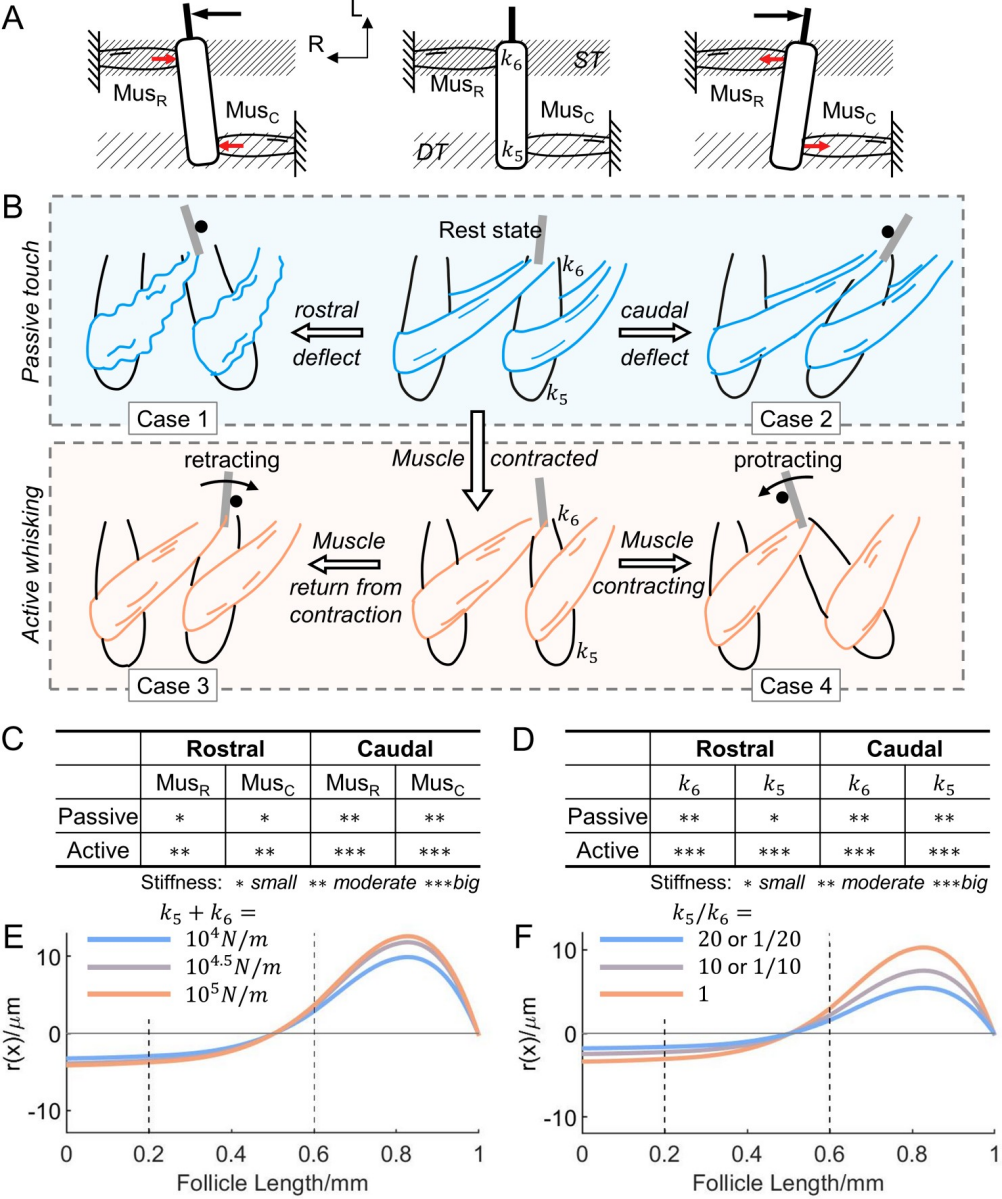
Caudal deflection and actuated intrinsic muscles result in increased whisker displacement. **(A)** The ***middle*** panel illustrates the follicle at rest, with superficial tissue (ST) indicated by thin hatching and deep tissue (DT) indicated by coarse hatching. Illustrations in ***left and right*** panels show the directions of the reaction forces on the vibrissa when the whisker is deflected rostrally and caudally, respectively. The external force is in black, and the reaction forces are in red. L: lateral; R: rostral; Mus_R_: rostral intrinsic muscle; Mus_C_: caudal intrinsic muscle. **(B)** Illustrations show muscle behavior in four different cases. Each illustration shows two follicles and the connecting intrinsic muscles. The caudal of the two whiskers is deflected in either the rostral direction (left column) or caudal direction (right column), during both passive touch (blue box) and active whisking (orange box). Muscles are defined to be completely relaxed (blue) during passive touch, and actuated (orange) during active whisking. During passive touch, the whisker is deflected by an external peg, marked by a black circle. During active whisking, the caudal follicle retracts/protracts against a peg, so that the whisker is deflected rostrally/caudally. The locations of two external springs, k_5_ and k_6_, are indicated in the two middle panels. **(C)** A table qualitatively summarizes intrinsic muscle stiffness for different cases of muscle activation and deflection direction. Asterisks indicate the ordinal (not proportionally scaled) magnitudes of muscle stiffness. **(D)** A table qualitatively summarizes k_5_ and k_6_ stiffness for different cases of muscle activation and deflection direction. Asterisks indicate the ordinal (not proportionally scaled) magnitudes of muscle stiffness. **(E)** The change in relative displacement r(x) under different overall external spring stiffness (k_5_+k_6_). The overall stiffness increases logarithmically from 10^4^N/m (blue) to 10^5^N/m (orange). A stiffer external support results in bigger r(x). **(F)** The identical plot as (E), but with different external spring stiffness ratios (k_5_/k_6_). As the ratio shifts from unbalanced (blue) to balanced (orange), larger r(x) is observed at the RS level.

**Fig 8:**
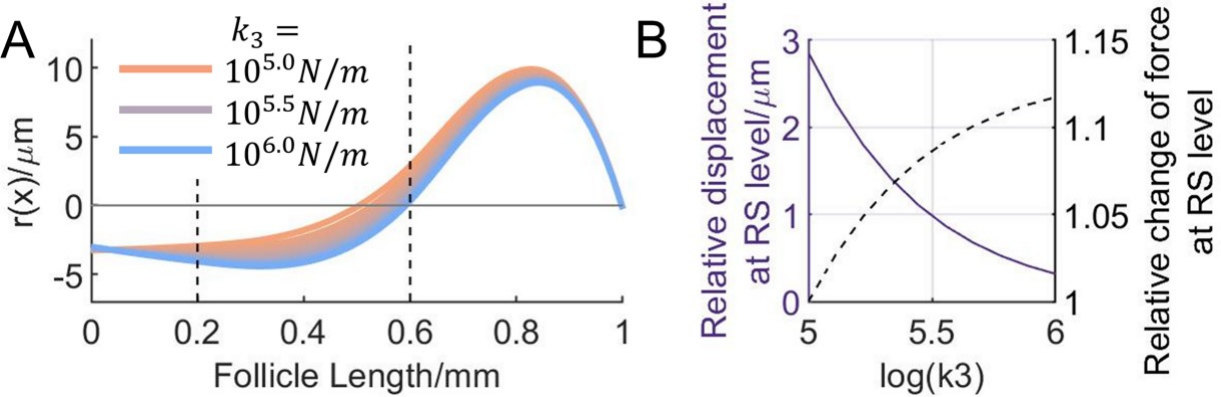
Increasing blood pressure causes decreased whisker displacement, but higher internal forces at the RS level. (A) The change of r(x) as the hydrostatic pressure in the RS is simulated to increase by increasing k_3_. From orange to blue, the value of k_3_ increases logarithmically from 10^5^ to10^6^N/m. With higher blood pressure, r(x) in the RS region decreases. (B) The change in the relative displacement and the force at the RS level as the blood pressure increase. Left y-axis, solid line: relative displacement at the RS level, r(x)|_x = 0.6mm_. Right y-axis, dashed line: the relative change of the internal force at the RS level from base state (k_3_ = 10^5^N/m). The force is given by k_3_∙r(x)|_x = 0.6mm_. As the blood pressure increases, the relative displacement at the RS level drops, whereas the force increases.

The next sections of this work describe the effects of changing the spring stiffnesses outside the follicle, assuming the S_1_- and S_2_-shaped profiles deemed feasible space in Fig 6. However, it is important to note that the same analysis (i.e., the results shown in Figs 7 and 8) would equally well hold for C-shaped profiles.

### Deformations of the whisker in the follicle are predicted to be larger during active whisking than during passive whisker displacement

All previous simulations have assumed a fixed stiffness outside the follicle, established by the springs k_5_ and k_6_. Their sum k_5_+k_6_ has been fixed at 10^4^N/m, reflecting the total stiffness outside the follicle. As explained earlier in Results, their ratio has been fixed at k_6_/k_5_ = 7/3, reflecting the relatively higher stiffness near the skin than deeper in the tissue.

However, we noted that these stiffnesses will change depending on whether the muscles are relaxed or contracted. We therefore simulated how the whisker displacement in the follicle would change between a deflection delivered passively versus a deflection generated by active whisking. We emphasize that the results of this section reveal qualitative trends only, and give an approximate indication of how deformations of the whisker will change based on behavioral conditions. To simulate the differences between passive and active deflection required five steps.

*Step 1) Notice that stiffness outside the follicle depends not only on the intrinsic muscles*, *but also on surrounding tissue*, *including the skin*, *surrounding collagenous extracellular matrix*, *tendons*, *and the extrinsic musculature*. *Therefore*, *both the total stiffness and the balance of stiffnesses on the two ends of the follicle will influence follicle motion*. The central panel of Fig 7A schematizes the two intrinsic muscles that connect to a typical follicle, as well as the surrounding superficial and deep connective and extrinsic muscle tissue. Inspection of this central panel indicates that the stiffness of the springs k_5_ and k_6_ will depend on the surrounding tissue as well as the muscles. In addition, the motion of the follicle will be determined both by the total stiffness of the springs k_5_ and k_6_ (their sum), as well as the relative strengths (their ratio). Notice also that tissue is not schematized in any other diagrams in Fig 7 for visual clarity. In the following steps, steps 2 through 4 neglect the tissue (focusing only on the muscles), and step 5 then adds its effects back in.

*Step 2) Analyze intrinsic muscle stiffness (neglect tissue)*: *notice that the direction of deflection determines whether the intrinsic muscles compress or lengthen*. The left and right panels of Fig 7A indicate the direction of reaction forces exerted by the muscles on the follicle when the vibrissa is deflected rostrally (left panel) and caudally (right panel). In these diagrams, all forces are indicated to act horizontally for visual simplicity, but a similar analysis holds if the force contains a vertical component. When the whisker is deflected rostrally, the intrinsic muscles on both sides of the follicle push to support the follicle. Consequently, both muscles are in compression. In contrast, when the whisker is deflected caudally, the intrinsic muscles on both sides of the follicle pull it in opposite directions and both muscles are stretched (they are in tension). Note that the schematics in Fig 7A are free body diagrams, so the reaction forces shown are independent of the displacement of adjacent follicles.

*Step 3) Analyze intrinsic muscle stiffness (neglect tissue): apply the analysis of Fig 7A to more realistic schematics.* Fig 7B schematizes four cases of whisker deflection, for both passive and active touch. In all schematics, two neighboring follicles in the same row are shown, serially connected by intrinsic muscles [50], and the caudal whisker is assumed to be deflected. In *passive touch*, the muscles are not contracted, and the whisker is assumed to be deflected by a peg moving relative to the whisker. A rostral deflection will cause the muscle to compress (case 1), while a caudal external deflection will cause the muscle to lengthen (case 2). In *active touch*, the muscles are assumed to be contracting so that the caudal follicle protracts or retracts the whisker against a peg. If the whisker makes contact with an object during retraction (case 3), the muscle will be passively lengthening, and the object will cause the muscle to compress. If the whisker makes contact with an object during protraction (case 4), the muscles are contracting, and the object will cause the muscles to lengthen. Note that case 4 also includes the scenario in which an animal holds its whiskers stiff but does not actively whisk; this situation often occurs when a rodent runs along a wall. In accordance with the quasi-static assumptions of this work, actively holding the whisker at a fixed position is equivalent to an instant during active protraction.

*Step 4) Analyze intrinsic muscle stiffness (neglect tissue): the four cases of Fig 7B are associated with different mechanical properties.* Contracted muscles and passively relaxed muscles have different spring stiffness when they are compressed or lengthened by an external force from “natural” or resting length [51]. Fig 7C qualitatively summarizes the intrinsic muscle spring stiffnesses for the four cases shown in Fig 7B. Specifically, in case 1, a rostral deflection will compress the muscles and be associated with low stiffness (single asterisk). In case 2, a caudal deflection will lengthen the relaxed muscles from their resting lengths and be associated with moderate muscle stiffness due to connective tissue (double asterisks). In case 3, although muscles cannot actively resist a compression force, active muscle fibers are likely still cross-linked, contributing to larger stiffness and force resistance (double asterisks). In case 4, the contracted muscles will actively resist the tensile force, resulting in high stiffness (triple asterisks).

*Step 5) Incorporate the effects of surrounding tissue*. As indicated in Step 1, the external springs k_5_ and k_6_ represent the combined stiffness of intrinsic muscles and connective and muscle tissue external to the follicle. Steps 2 through 4 have analyzed intrinsic muscle stiffness only, while neglecting the tissue, and the qualitative stiffness estimates of Fig 7C. Fig 7D now qualitatively depicts stiffness after incorporating the effects of the surrounding tissue.

In case 1, the intrinsic muscles are relaxed and compressed to be shorter than their resting lengths, so they exert only weak reaction forces, therefore the stiffness contribution of the connective tissue around the follicle is likely to be significant. Specifically, during an external deflection, the collagen layer and the keratinous skin epidermis will anchor the follicle near its apex, while the deeper end of the follicle will displace. Therefore, in Fig 7D, the stiffness of k_6_ (near the apex) is assigned double asterisks, while k_5_ (near the base) remains a single asterisk. For the other cases, the contribution of connective tissue to the overall stiffness is not as significant compared to that of the intrinsic muscles. However, for cases 3 and 4, when the rat is actively whisking, a complex of extrinsic muscles is activated and help hold the follicles firmly. These extrinsic muscles insert into the mystacial pad from different directions, in both superficial and deep layers. Although different muscle groups are activated at different phases during whisking, a relatively stiffer support is offered by at least one group of muscles at each phase. Therefore, the overall stiffness for cases 3 and 4 in Fig 7D is assigned triple asterisks.

In order to investigate how the whisker deformation profile will be different during active whisking compared to passive touch, without having to fix a single stiffness condition for each, we examined the qualitative trends from passive touch to active whisking by independently changing the overall stiffness (k_5_+k_6_) and the balance (k_5_/k_6_) of the external support. These degrees of freedom relating to tissues and muscles can be disentangled into independent variables (k_5_+k_6_ and k_5_/k_6_) due to the intrinsic linear property of the system, which frees us from having to consider any coupling effects.

We first looked at the overall stiffness of the external support. The overall stiffness is modeled by the summation of the external spring constants (k_5_+k_6_). Fig 7E shows how the relative displacement r(x) changes under different overall stiffness. As expected, stiffer external support in general prevents the follicle from rotating. Consequently, for an imposed external deflection angle, r(x) must increase in magnitude to compensate for the small rotation of the follicle. Specifically, r(x)|_x = 0.6mm_, increases from 2.97μm to 3.66μm (123.10% of its original), when the overall stiffness increases from 10^4^ to 10^5^N/m. The result of the simulation indicates that active muscles of high stiffness result in larger whisker deformation followed by larger tissue displacement internal to the follicle.

We next looked at the balance of external support. Fig 7F shows the relative displacement r(x) for different external spring ratios (k_5_/k_6_), with the value of the sum held constant. This result shows that when external support to the follicle is more balanced, the relative displacement of the whisker within the follicle increases. This effect occurs because the follicle itself rotates less when the whisker is deflected (absolute displacement not shown), meaning that the whisker shaft itself must bend more to accommodate the externally imposed movement. Specifically, r(x)|_x = 0.6mm_ decreases from 2.97μm to 1.57μm (52.94% of its original), by switching from balanced support (k_5_/k_6_ = 1) to unbalanced support (k_5_/k_6_ = 1/20 or 20). The result suggests that more balanced external support will also result in larger whisker deformation, hence larger tissue displacement internal to the follicle.

The results suggest that these two independent variables both facilitate tactile sensitivity. This leads to the combined effect of muscle being actuated quite clear: actuated muscle leads to larger tissue displacement than unactuated muscle, and presumably higher tactile sensitivity. We emphasize that although the analysis of Fig 7 has assumed an S-shaped profile, all results would hold equally well for any of the C-shaped profiles shown in Fig 6.

### Increased blood pressure in the RS results in larger whisker deformation

The blood flow within the follicle at the RS is regulated by the autonomic nervous system [52]. It has long been postulated that this regulating blood pressure could stabilize mechanical properties of the vibrissa [33–36], mediate tactile sensing resolution for some slowly-adapting receptors [33,34], allowing animals to have different perceptual sensitivities as needed. However, the relationship between blood pressure in the RS and sensation is difficult to validate *in vivo*. In our model, a change of hydrostatic (blood) pressure in the RS is simulated by changing the value for k_3_, thus allowing us to estimate the effects of changing pressure on whisker deformation within the follicle.

Fig 8A shows that the relative whisker displacement r(x) decreases at the RS level as k_3_ increases, modeling increasing hydrostatic pressure. Specifically, r(x)|_x = 0.6mm_ drops from 2.86μm to 0.32μm (11.17% of its original), when k_3_ increases from 10^5^ to10^6^N/m. The general deformation profiles remain similar.

Mechanoreceptor response is largely associated with the tissue deformation in that small area which surrounds it. In the previous section, the whisker displacement is itself a good indicator of tissue deformation. Here, due to the inflated and stiffened RS structure, the whisker displacement alone offers only incomplete information about tissue deformation. Therefore, we made a preliminary assessment of tissue deformation by looking at the internal force exerted on the tissue at the RS level. This force is defined as the one which occurs between the RS and the whisker shaft, and is simply given by multiplying the tissue stiffness (k_3_) and the relative whisker displacement at the RS level (r(x)|_x = 0.6mm_). Fig 8B shows how changes in blood pressure affect relative whisker displacement and the internal force at the RS level, with higher pressure leading to smaller displacement. As blood pressure increases, although whisker displacement decreases, the product of stiffness and displacement increases. In other words, the internal force becomes larger. This force is important if we assume the amount of tissue deformation is related to the force that is applied to it. The implications for mechanosensitivity will be further described in the *Discussion*. Once more, we emphasize that although the analysis of Fig 7 has assumed an S shaped profile, all results would hold equally well for any of the C-shaped profiles shown in Fig 6.

## Discussion

We have developed a low-dimensional mechanical model that predicts the shape of the whisker in the follicle as it pushes and pulls against mechanosensitive regions. The model can reveal only qualitative deformation profiles; the absolute magnitudes of displacements and forces should not be taken as precise predictions. The number of model parameters was chosen to be commensurate with available biological data. The model is based on a quasi-static analysis and in its present form cannot be directly used to predict dynamic effects. Although we anticipate many future improvements, the present model serves to bracket the range of possible internal states and describes consequences for sensory receptor activation and the regulation of tactile sensitivity.

### The simulation predicts whisker displacement in all areas within the FSC, but mechanoreceptor responses will depend also on local tissue stiffness

Four springs were used to model the stiffnesses within the follicle. Although the spring stiffness distribution reflects tissue stiffness, they are not equivalent: tissue stiffness is more traditionally represented by Young’s modulus. However, approximating Young’s modulus would require us to assume locations for the springs at a higher spatial resolution than appropriate given the available biological data. Instead, the springs’ locations near the HB, CS, RS, and RRC were chosen because qualitative observation of follicle anatomy (e.g., Fig 4) indicated that tissue material properties are likely to be similar within each of the three partitioned regions. Future work will explore the effects of adding additional springs to the model.

Although adding more springs would add resolution to the model, we have confidence in the general shapes of the profiles, as they persist for a wide range of parameter combinations. Consistent with previous hypotheses for whisker deformation in the follicle [36,48], the model predicts that the whisker will pivot about a fulcrum near its apex. After fixing the value of the spring near the follicle entrance, varying the values of the three remaining internal springs revealed three distinct feasible whisker shapes: C-shaped, S1-shaped, and S2-shaped (Figs 5 and 6). The primary reduction in feasible profiles resulted from limiting deformation amplitude at the RS level to 4.8 microns (Fig 6). This constraint was based on results of an *ex vivo* imaging experiment [37] and the consequences of relaxing this restriction will be explored in a later section of the Discussion.

Regardless of whether the profile is C-shaped or S-shaped, larger whisker displacements will result in larger tissue deformation. However, displacement alone is not enough to predict the forces that the whisker will exert on mechanoreceptors: an estimate of local tissue stiffness is required. To gain intuition for the relative roles of whisker deformation and local stiffness, imagine a room in which a balloon filled with air is hung by a string from the center of the ceiling. If you push on the balloon, it will simply bump away from you. You will not be able to deform the balloon’s shape because there is nothing holding it in place as you push on it. Now imagine the same balloon but hung from the ceiling very close to a wall. When you push on the balloon it will compress against the wall. Because the wall is stiff, the balloon will deform.

Similarly, the identical whisker deformation in the follicle could lead to very different mechanoreceptor responses, depending on the local tissue stiffness. Because the present model does not address the microstructure of the tissue in which the mechanoreceptors are embedded, the whisker deformation profiles do not directly map to how the mechanoreceptors respond. The whisker displacement can serve only as an indirect predictor of internal tissue deformation.

### What are the mechanisms by which local tissue stiffness can be modulated?

Because so much of the mechanoreceptor response will depend on local stiffness, the present work has also investigated tissue stiffness in different regions of the follicle and how it might be modulated. The anatomical analysis of Fig 4 indicates that stiffer tissue is found closer to the follicle entrance, near the mechanoreceptor rich regions. This finding indicates that mechanoreceptors in these regions are compressed against relatively stiff tissue, compared to deeper regions, allowing richer tactile sensitivity and resolution.

Notably, the spring that has the largest influence on whisker deformation (k3) is located at the RS, the most richly innervated region of the follicle. Blood pressure variation in the RS has previously been hypothesized to help regulate tactile sensitivity during active whisking [33–36]. Because the follicle walls are stiff, the follicle capsule is well approximated as a system with a fixed volume. Increasing the volume of fluid in the follicle will increase the hydrostatic pressure and hence the stiffness. In agreement with this hypothesis, the results of the present work indicate that a stiffer RS will result in decreased radial whisker displacement, and increased force in the radial direction (Fig 8). This radial force is likely to have a strong effect on slowly-adapting RS-Merkel cells [49,53], making them especially good for sustained pressure sensing. The increase in radial force might also serve to increase displacement of mechanoreceptors in the other two dimensions.

Tissue stiffness is also strongly influenced by the state of the muscles surrounding the follicle, and these effects are discussed further below when considering the differences between passive touch and active whisking.

### Mappings from the whisker deformation profile to the mechanoreceptor response

**Fig 9:**
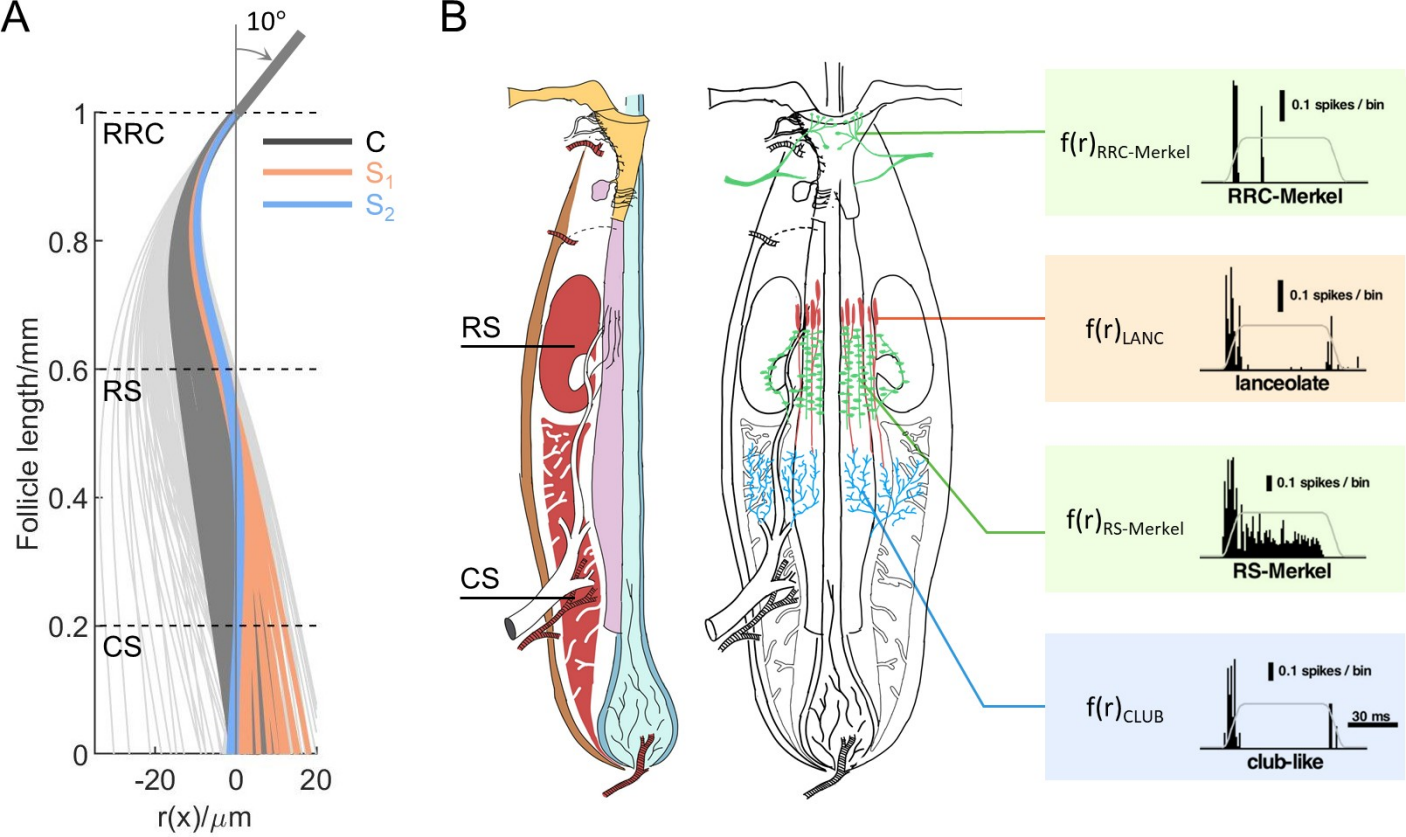
Schematics indicate how whisker deformation profiles map to the responses of different subtypes of mechanoreceptors **(A)** The deformation profiles from Fig 6 are replicated here. Although the S-shaped profiles (blue and orange) are the focus of the present work, some of the C-shape profiles (dark gray) would be possible if constraints based on experimental data were relaxed (see text for details) **(B)** Left: The locations of the two blood sinuses (left panel) and the different subtypes of mechanoreceptors are shown vertically aligned with the deformation profiles in (A). Middle: Four major types of mechanoreceptors are shown: RRC-Merkel endings (green), Lanceolate endings (red), RS-Merkel (green), and Club-like endings (blue). Right: Mappings to each of the mechanoreceptor types are indicated as boxes. A time series of whisker deformation profiles r(x, t) yields different responses f(r) for each mechanoreceptor type. The responses of the different subtypes are represented by peri-stimulus time histograms (PSTHs), adapted from Furuta et al. 2020 [49]. In this example, f(r) is the mapping f: r(x, t)→PSTH.

Although the present model cannot predict specific mechanoreceptor cell-membrane displacements, it does allow us to begin to form a mapping between the whisker deformation profile and neural activity of the mechanoreceptors. The same external deflection will lead to very different neural responses by different subtypes of mechanoreceptors at different locations. To begin to consider the consequences of whisker deformation on mechanoreceptor responses, the profiles of Fig 6 are replicated in Fig 9A, and aligned with two schematics of the follicle that emphasize the locations of the blood sinuses and the different mechanoreceptor types (the left two panels of Fig 9B).

It is notable that the S-shaped profiles do not exhibit maximal whisker deformation near the RS, where most of the mechanoreceptors are located. As described in *Results*, these S-shaped profiles occur because the whisker shaft displacement near the RS was limited to 4.8μm, the norm of the average, 3D tissue displacement observed experimentally when the whisker is deflected by 10° [37]. The dark gray profiles of Fig 9A show the consequence of relaxing this upper bound to 12μm, which is the norm of the maximal tissue displacement recorded in the same experiment. Unsurprisingly, with more flexibility available near the RS, the deformation profiles approach C-shapes. Although an S-shape may seem unintuitive, it is possible that such a profile could help avoid a saturation effect during large deflections. In other words, increasing stiffness near the RS would allow larger external forces to be imposed, without generating excessive displacement of the whisker inside the follicle. Since we cannot say with absolute certainty which profile may occur (perhaps both), it is important to note that the results of both Figs 7 and 8 in the present work are equally valid for both S-shaped and C-shaped profiles. Until more experiments are performed, the S-shaped profiles are most consistent with the available biological data.

Regardless of whether the profiles are C-shaped or S-shaped, Fig 9A also clearly indicates that more lateral to and at the level of the RS, the whisker will displace in the direction opposite that of external whisker deflection. This result is intriguing given that recent work has shown that both RS-Merkel and RRC-Merkel cells respond most strongly when the external whisker is deflected in the same direction as the mechanoreceptors [49]. Previous studies have shown that the Merkel cell complex with slowly adapting type I afferents responds specifically to tissue compression [54,55], and that the Piezo2 channels in Merkel cells respond to positive pressure (compression) only [56,57].

To explain the responses of the RRC-Merkel cells, we note that they are located on the fringes of the FSC. When deflected, these cells are likely to be compressed in the same direction as the deflection; with little or no effect on the other side of the FSC. Explaining the observed responses of the RS-Merkel cells is more challenging. The epithelial tissue, glassy membrane, and connective tissue at the RS level is not connected to any hard tissue (e.g., the follicle wall), and deformation will affect both sides of the FSC. The RS-Merkel cells themselves are located between the glassy membrane and the epithelial sheath [49]. These factors, along with others, will lead to complicated interactions between shaft displacement and Merkel cell membrane displacement. The relationship between the compression/stretch of the tissue and the neural activity of RS-Merkel cells is unclear. The difficulty in determining such relationships underlies the importance and usefulness of seeking a mapping from one to the other.

Fig 9B illustrates an example of such mapping from a time series of whisker deformation profiles (r(x, t)) to neural activities of different subtypes of mechanoreceptors. Previous work has characterized how different mechanoreceptor subtypes respond to a ramp-and-hold stimulation in one direction [49]. A time series of deformation profiles can be simulated by applying the same stimulus to the model of the whisker. In this way, a mapping f: r(x, t)→neural activity can be generated for each individual subtype. We anticipate that future model improvements will yield a richer description of whisker displacement in all dimensions, which is likely to be particularly important for lanceolate and club-like endings. A 3D model will not only allow analysis of longitudinal and polar tissue deformation, but also improve the mapping to neural activity.

### Generalization from passive deflections to active whisking

In the most general terms, the present model suggests that the deformation profile of the whisker will stay qualitatively the same between passive touch and active whisking: the same group(s) of mechanoreceptors will respond when the whisker is deflected in the same direction under both conditions. This consistency is advantageous to a whisker-specialist animal that must interpret whisker signals during both active and passive conditions, because it is easier to interpret a system’s responses if it does not change with behavioral state.

However, we emphasize that the model treats both protraction and retraction as quasistatic processes, and thus the present work can predict deformation of the whisker in the follicle only in response to external bending. Examination of dynamic effects such as vibrations or collisions will require the addition of dampers to model more transient responses. The present model cannot predict the whisker’s time-varying response during texture exploration [58–64], during an airflow stimulus [65], or the reafferent signals during non-contact (“free-air”) whisking [53,66–68].

Despite these limitations, the present model can be used to investigate the differences between an external tactile stimulus delivered when the muscles are relaxed versus contracted (Fig 7). When the external springs that represent the external tissue and muscle stiffness are stiffer, as during active whisking, tactile sensitivity is enhanced. More interestingly, the model predicts that tactile sensitivity will depend on the balance of superficial and deep muscles. During a rostral deflection, muscle support of the follicle is more unbalanced than during a caudal deflection, and tactile sensitivity is reduced (Fig 7F). Thus overall, the simulations suggest that muscle actuation in general, as well as caudal external deflection, both serve to increase mechanosensitivity, and these are precisely the conditions that obtain during active touch.

Recent behavioral work has shown that rats typically prolong the total duration of contacts significantly in a whisk cycle [69,70]. This is enough time required for vibrations to damp after collision with the object [71] and the whisker will experience quasistatic bending. The present model can be used to begin to understand how the whisker deforms in the follicle under these conditions, and how mechanoreceptors may respond during these temporal “windows” of whisking behavior. The current work thus paves the way for future studies to more completely characterize internal whisker follicle mechanics to investigate the responses of sub-populations of mechanoreceptors and other specialized compartments within the follicle.

## Supporting information

S1 Fig The deformation profile of the whisker is determined by tissue stiffness both inside and outside the follicle. Three hypothetical cases for how the whisker-follicle complex could deform and rotate in response to an external force F. The whisker follicle complex is shown in its resting position in the schematic labeled “rest,” and the three cases show its final position and shape after the force has been applied. In case 1, the tissue outside the follicle is extremely stiff. As a result, the whisker bends without little or no follicle rotation. In case 2, the tissue inside the follicle is extremely stiff. The imposed force causes the follicle to move as a whole and the whisker bends very little. In case 3, the tissue stiffness surrounding the follicle is moderate, so the whisker bends, and the follicle also rotates. In the present work, simulations generate the shapes of case 3, but only relative displacements (the x-y reference frame in red) are reported and analyzed in Results.
(TIF)
