# Supplementary figures and images for: Constraints on the deformation of the vibrissa within the follicle
Source: PLoS Comput Biol. 2021 Apr 1;17(4):e1007887. doi: 10.1371/journal.pcbi.1007887 (PMC8016108; doi:10.1371/journal.pcbi.1007887)

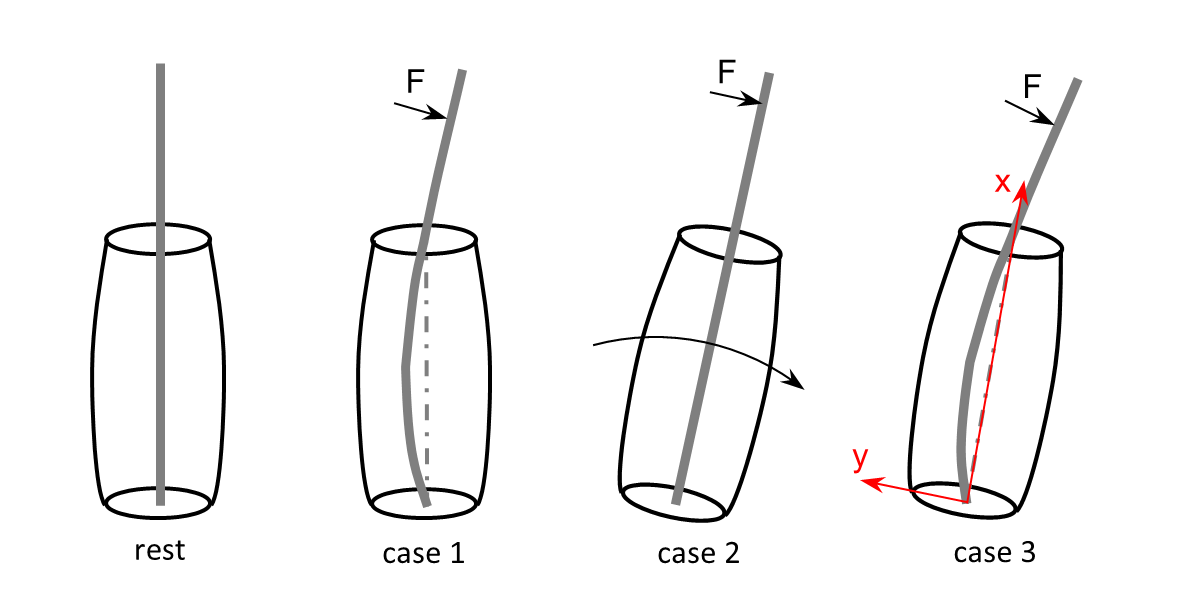

Supplement: S1 Fig — Three hypothetical cases for how the whisker-follicle complex could deform and rotate in response to an external force F. The whisker follicle complex is shown in its resting position in the schematic labeled “rest,” and the three cases show its final position and shape after the force has been applied. In case 1, the tissue outside the follicle is extremely stiff. As a result, the whisker bends without little or no follicle rotation. In case 2, the tissue inside the follicle is extremely stiff. The imposed force causes the follicle to move as a whole and the whisker bends very little. In case 3, the tissue stiffness surrounding the follicle is moderate, so the whisker bends, and the follicle also rotates. In the present work, simulations generate the shapes of case 3, but only relative displacements (the x-y reference frame in red) are reported and analyzed in Results. (TIF) [file pcbi.1007887.s001.tif]
